# Supplementary material for: Sulfo-Gambierones, Two New Analogs of Gambierone Produced by Gambierdiscus excentricus
Source: Mar Drugs. 2021 Nov 24;19(12):657. doi: 10.3390/md19120657 (PMC8703632; doi:10.3390/md19120657)
Supplement: Supplementary file 1 [file marinedrugs-19-00657-s001.zip › marinedrugs-1459038-supplementary.pdf]

marine drugs

Article Supplementary

Sulfo-Gambierones, Two New Analogs of Gambierone Produced by *Gambierdiscus excentricus*

Thomas Yon [1*], Manoëlla Sibat [1], Elise Robert [1], Korian Lhaute[1], William C. Holland [2], R. Wayne Litaker [3], Samuel Bertrand [4,5], Philipp Hess [1,*] and Damien Réveillon [1]

[1] Ifremer, DYNECO, Laboratoire Phycotoxines, F-44000, Nantes, France; manoella.sibat@ifremer.fr (M.S.); Elise.Robert@ifremer.fr (E.R.); Korian.Lhaute@ifremer.fr (K.L.); damien.reveillon@ifremer.fr (D.R.)
[2] NOAA; National Centers for Coastal Ocean Science, National Ocean Service, Beaufort Laboratory, Beaufort, NC 28516, USA; chris.holland@noaa.gov
[3] CSS, Inc. Under Contract to National Oceanic and Atmospheric Administration, National Ocean Service, National Centers for Coastal Ocean Science, Beaufort, NC 28516, USA; wayne.r.litaker@noaa.gov
[4] Nantes Université, Institut des Substances et Organismes de la Mer, ISOMER, UR 2160, F-44000 Nantes, France ; samuel.bertrand@univ-nantes.fr
[5] ThalassOMICS Metabolomics Facility, Plateforme Corsaire, Biogenouest, Nantes, France
* Correspondence: Thomas.yon@ifremer.fr (T.Y.); Philipp.hess@ifremer.fr (P.H.)

Table S1. MRM transition used on the LRMS system in negative ionization mode

Compound name	Transitions (precursor ion/product ion)	Ion formula
MTX1	1689.8/1689.8	$[M-2H]^{2-}/[M-2H]^{2-}$
	1689.8/96.9	$[M-2H]^{2-}/[HOSO_3]^{-}$
	1126.2/1126.2	$[M-3H]^{3-}/[M-3H]^{3-}$
	1126.2/96.9	$[M-3H]^{3-}/[HOSO_3]^{-}$
MTX2	1637.8/1637.8	$[M-2H]^{2-}/[M-2H]^{2-}$
	1637.8/96.9	$[M-2H]^{2-}/[HOSO_3]^{-}$
	1091.8/96.9	$[M-3H]^{3-}/[HOSO_3]^{-}$
MTX4	1646.2/1646.2	$[M-2H]^{2-}/[M-2H]^{2-}$
	1646.2/96.9	$[M-2H]^{2-}/[HOSO_3]^{-}$
	1097.8/1097.8	$[M-3H]^{3-}/[M-3H]^{3-}$
	1097.8/96.9	$[M-3H]^{3-}/[HOSO_3]^{-}$
MTX Unknown1	1649.8/1649.8	
	1649.8/96.9	
MTX Unknown2	1641.8/1641.8	
	1641.8/96.9	
Gambierone	1023.52/1023.52	$[M-H]^{-}/[M-H]^{-}$
	1023.52/97.0	$[M-H]^{-}/[HOSO_3]^{-}$
	1023.52/899.4	
	1023.52/963.5	
44-methylgambierone	1037.512/1037.512	$[M-H]^{-}/[M-H]^{-}$
	1037.512/97.0	$[M-H]^{-}/[HOSO_3]^{-}$
	1037.512/899.5	
	1037.512/977.6	
Gambieroxide	1193.6/96.9	$[M-H]^{-}/[HOSO_3]^{-}$
	1193.6/987.6	
	1193.6/1193.6	$[M-H]^{-}/[M-H]^{-}$
Gambieric Acid A	1055.1/1037.1	$[M-H]^{-}/[M-H_2O-H]^{-}$
	1055.1/1055.1	$[M-H]^{-}/[M-H]^{-}$
Gambieric Acid B	1069.1/1069.1	
	1069.1/1051.1	
Gambieric Acid C	1183.7/1183.7	
	1183.7/1165.7	
Gambieric Acid D	1197.7/1197.7	
	1197.7/1179.7	

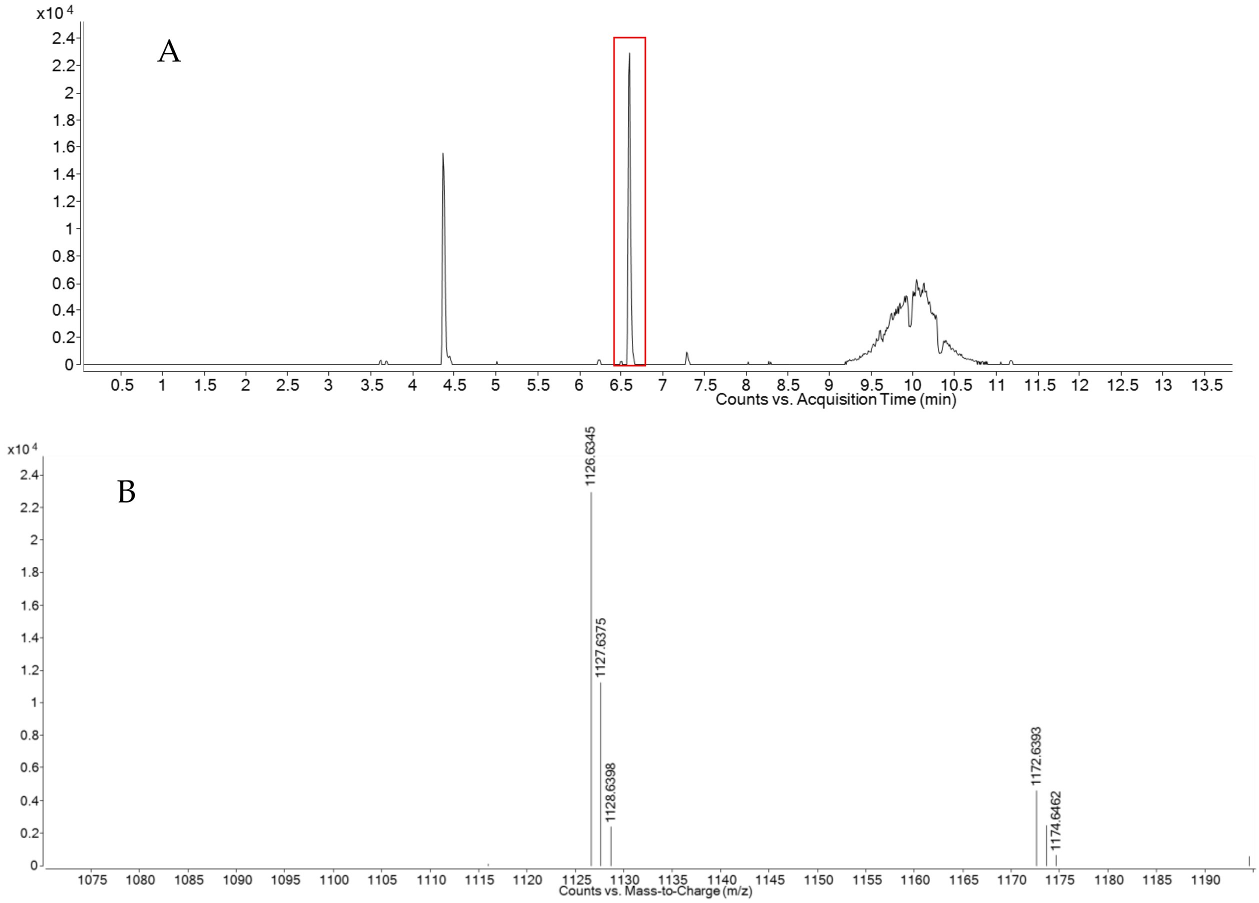

Figure S1. Extracted Ion Chromatogram (**A**) and zoom on full scan spectrum (**B**) corresponding to peak **(2)** presented in Figure 1 obtained with the method 2 with HRMS following m/z 1126.2 +/- 0.7

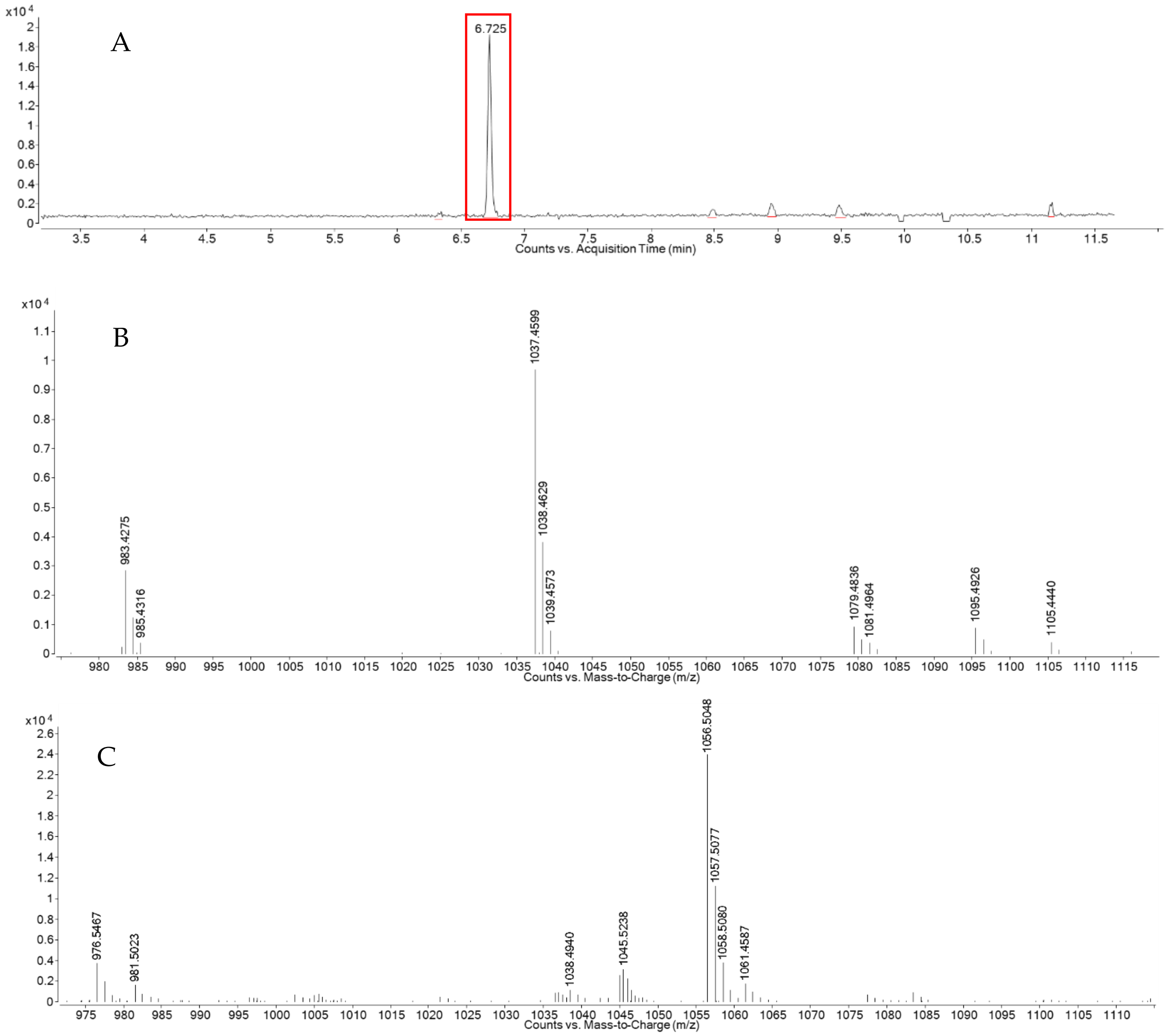

Figure S2. Extracted Ion Chromatogram (**A**) and zoom on full scan spectrum in ESI- (**B**) and ESI + (**C**) corresponding to peak **(3)** presented in Figure 1 obtained with the method 2 with HRMS fol-lowing *m/z* 1037.5 +/- 0.7

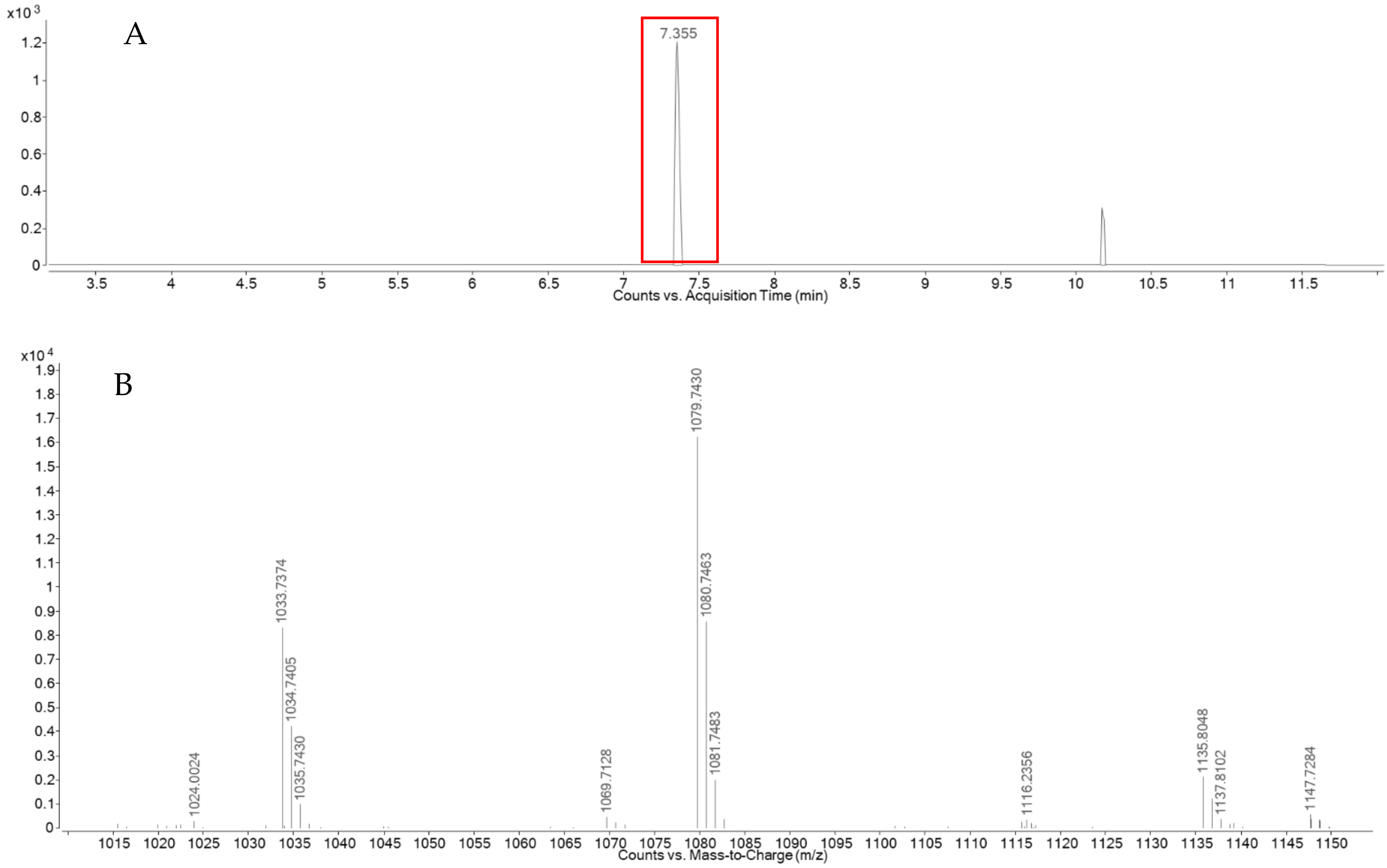

Figure S3. Extracted Ion Chromatogram (**A**) and zoom on full scan spectrum (**B**) corresponding to peak **(4)** presented in Figure 1 obtained with the method 2 with HRMS following *m*/*z* 1183.0 +/- 0.7

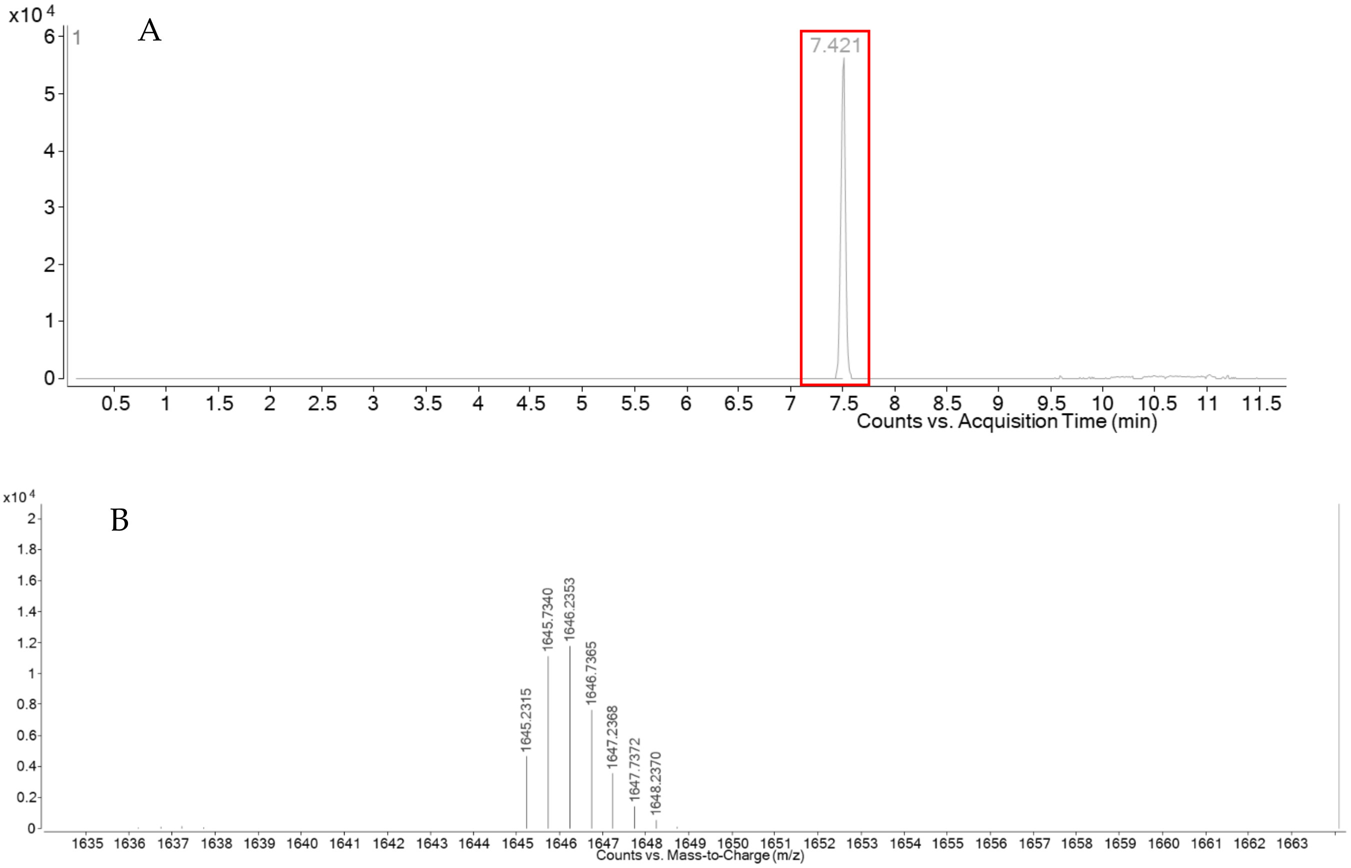

Figure S4. Extracted Ion Chromatogram (**A**) and zoom on full scan spectrum (**B**) corresponding to peak **(5)** presented in Figure 1 obtained with the method 2 with HRMS following *m*/*z* 1646.2 +/- 0.7

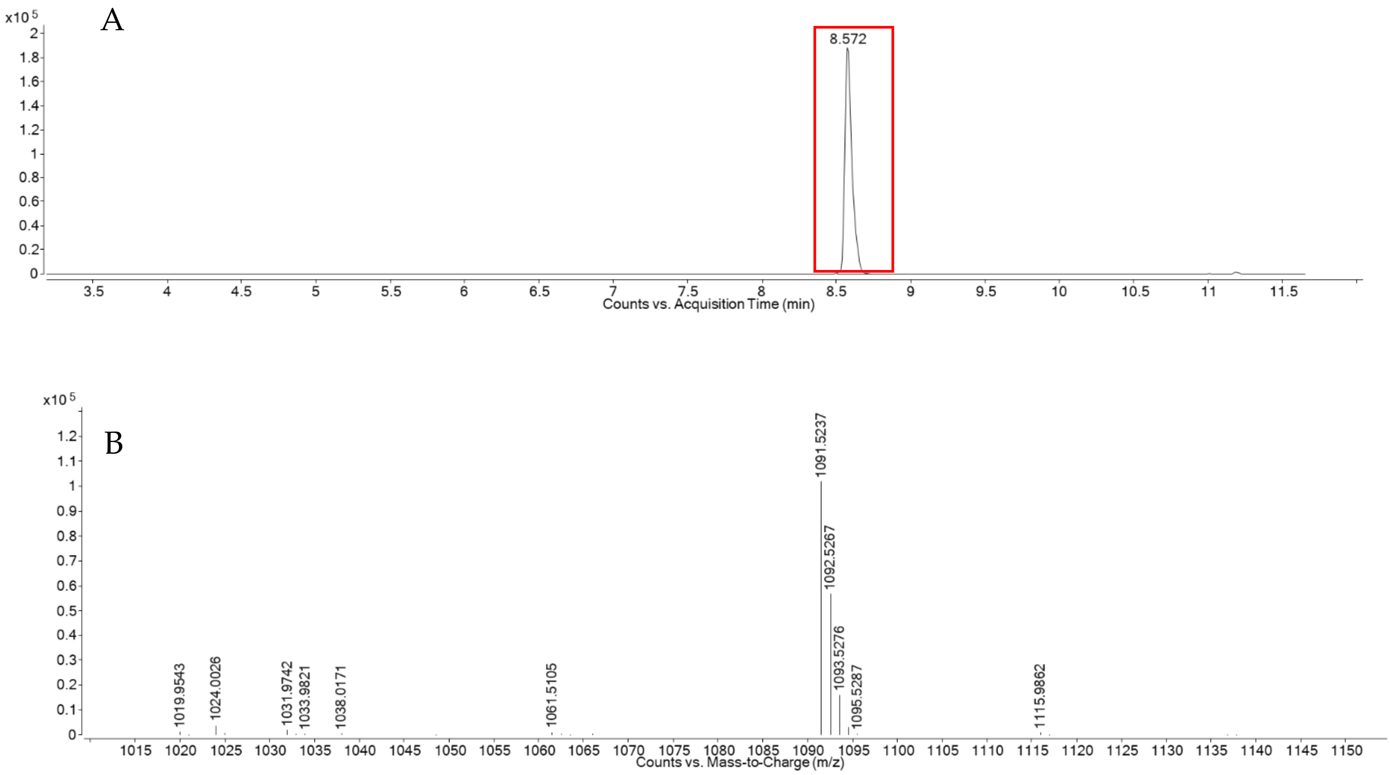

Figure S5. Extracted Ion Chromatogram (**A**) and zoom on full scan spectrum (**B**) corresponding to peak **(6)** presented in Figure 1 obtained with the method 2 with HRMS following *m*/*z* 1091.8 +/- 0.7

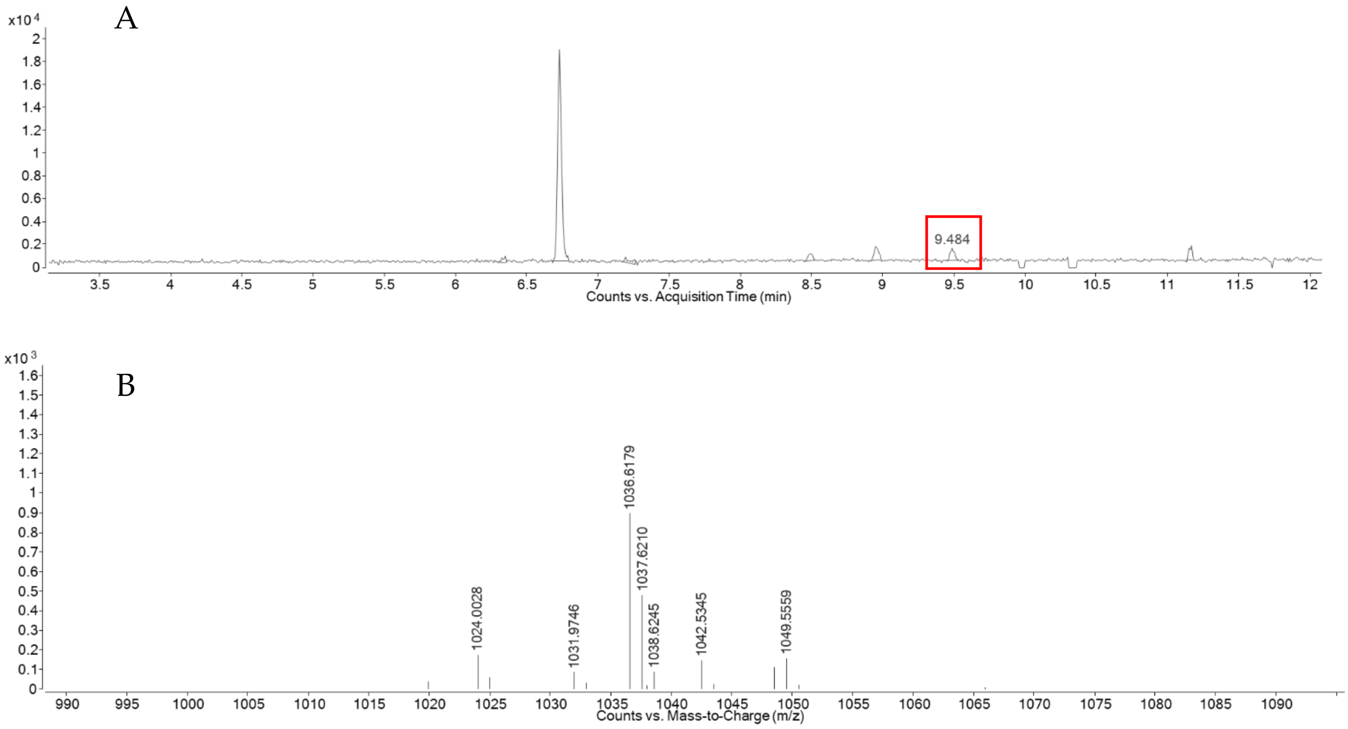

Figure S6. Extracted Ion Chromatogram (**A**) and zoom on full scan spectrum (**B**) corresponding to peak **(8)** presented in Figure 1 obtained with the method 2 with HRMS following *m/z* 1037.5 +/- 0.7

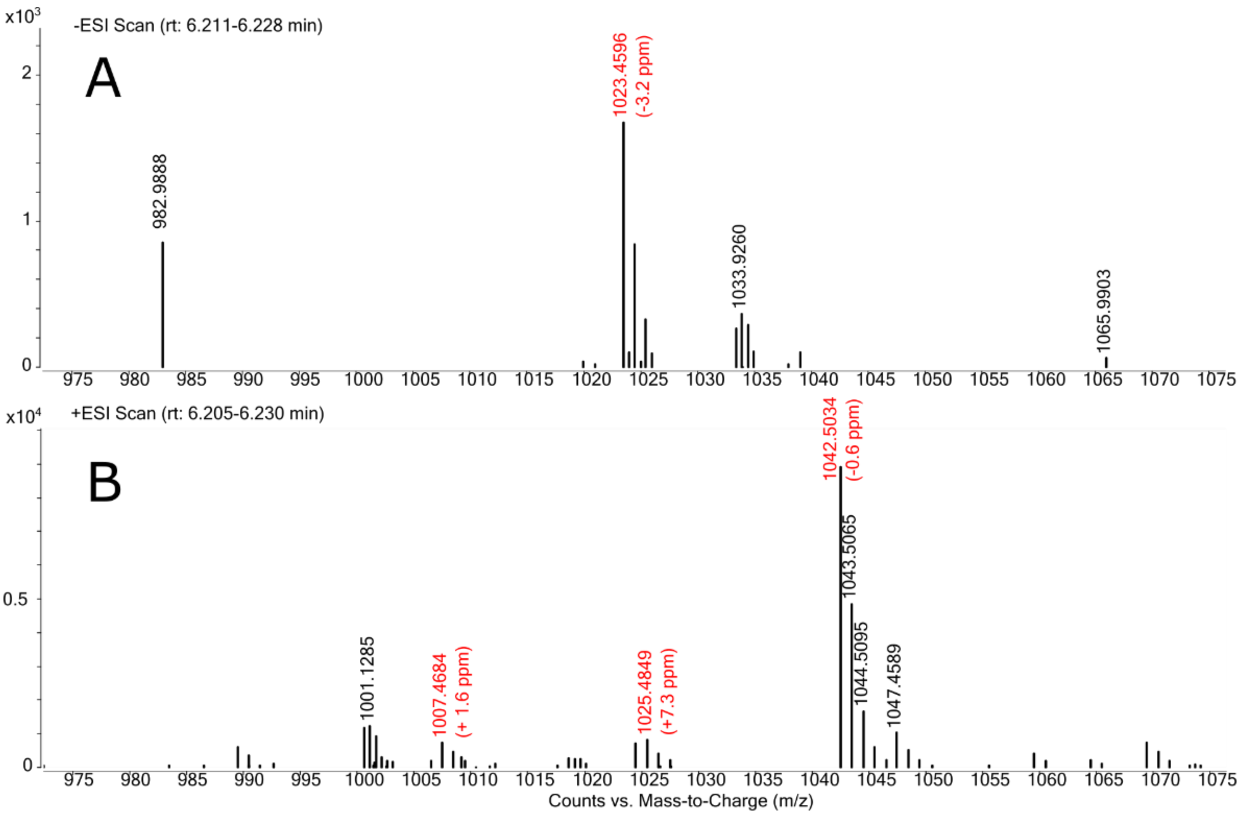

Figure S7. Zoom on the full scan (*m/z* 975-1075) corresponding to peak **(1)** presented in Figure 1 obtained with the method 2 at 6.2 min on the HRMS system in (**A**) negative mode and (**B**) positive mode.

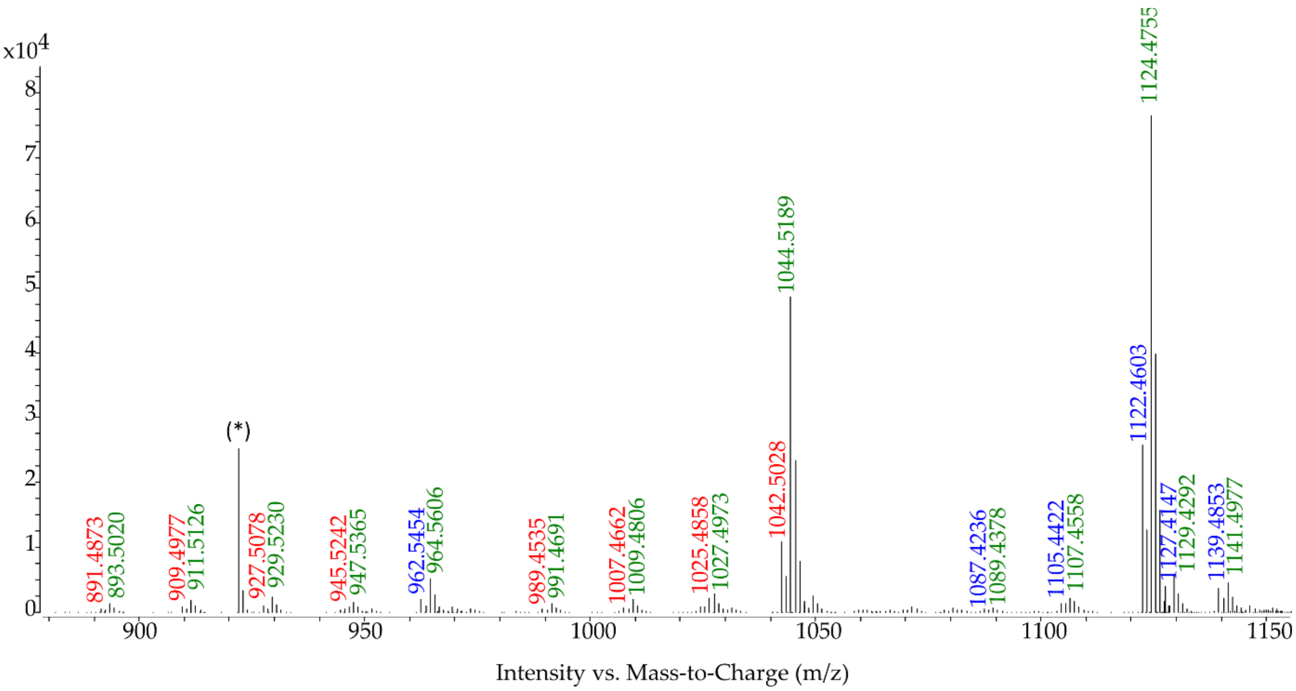

Figure S8. Zoom on the ESI⁺ full scan (*m/z* 880-1160) acquired with the method 3 on the chromatographic peak at 6.2 min obtained after injection of the concentrated pool of preparative-HPLC fractions (29.8 µg eq gambierone mL^{-1}). The ions already reported for gambierone are shown in red. Additional ions specific to sulfo-gambierone are shown in blue and ions related to the dihydro-sulfo-gambierone are shown in green. (*) reference mass.

Table S2. Ion species corresponding to the accurate monoisotopic *m*/*z* of sulfo-gambierone with mass differences (Δppm) between theoretical monoisotopic mass and measured *m*/*z*. Ions already reported in the literature for gambierone are shown in red, the proposed fragmentation pathway and corresponding ions are shown in blue and hypotheses and ions resulting from the modification of the structure compared to gambierone are shown in green.

Ion formula	Ion	**Theoritical monoisotopic mass**	**Measured *m*/*z***	Δ (ppm)
C51 H76 O22 S2 + NH_4^+	M+NH_4^+	**1122.4608**	**1122.4586**	-2.0
C51 H76 O22 S2 + H^+	M+H^+	**1105.4342**	**1105.4311**	-2.8
C51 H74 O21 S2+ H^+	M-H2O+H^+	**1087.4237**	**1087.4199**	-3.5
C51 H72 O20 S2+ H^+	M-2H2O+H^+	**1069.4131**	**1069.4065**	-6.2
C51 H76 O19 S+ NH_4^+	M-SO3+NH_4^+	**1042.504**	**1042.5026**	-1.3
C51 H76 O19 S+ H^+	M-SO3+H^+	**1025.4774**	**1025.4769**	-0.5
C51 H74 O18 S+ H^+	M-SO3-H2O+H^+	**1007.4668**	**1007.4654**	-1.4
C51 H72 O17 S+ H^+	M-SO3-2H2O+H^+	**989.4563**	**989.4523**	-4.0
C51 H70 O16 S+ H^+	M-SO3-3H2O+H^+	**971.4457**	**971.4436**	-2.2
C51 H76 O16 +NH_4^+	M-2SO3+NH_4^+	**962.5472**	**962.5446**	-2.7
C51 H76 O16+ H^+	M-2SO3+H^+	**945.5206**	**945.5185**	-2.2
C51 H74 O15+ NH_4^+	M-2SO3+NH_4^+	**944.5366**	**944.5343**	-2.4
C51 H75 $O15^+$	M-2SO3-H2O+H^+	**927.51**	**927.5079**	-2.3
C51 H73 $O14^+$	M-2SO3-2H2O+H^+	**909.4995**	**909.4964**	-3.4
C51 H71 $O13^+$	M-2SO3-3H2O+H^+	**891.4889**	**891.4872**	-1.9
C51 H69 $O12^+$	M-2SO3-4H2O+H^+	**873.4783**	**873.4754**	-3.3
C51 H67 $O11^+$	M-2SO3-5H2O+H^+	**855.4678**	**855.4614**	-7.5
C45 H67 $O14^+$		**831.4525**	**831.4507**	-2.2
C45 H65 $O13^+$		**813.442**	**813.4393**	-3.3
C45 H63 $O12^+$		**795.4314**	**795.4255**	-7.4
C45 H61 $O11^+$		**777.4208**	**777.4227**	2.4
C38 H55 O8 $^+$		**639.3891**	**639.3847**	-7.0
C38 H53 O7 $^+$		**621.3786**	**621.3803**	2.8
C38 H51 O6 $^+$		**603.3680**	**603.3649**	-5.2
C32 H45 O6 $^+$		**543.3316**	**543.3278**	-7.0
C38 H53 O7 $^+$		**525.3211**	**525.3197**	-2.6
C18 H25 O3 $^+$		**289.1798**	**289.1811**	4.4
C18 H23 $O2^+$		**271.1693**	**271.1712**	7.2
C15 H21 $O2^+$		**233.1536**	**233.1545**	3.8
C10 H11 O2 $^+$		**163.0754**	**163.0754**	0.0
C8 H11 O $^+$		**123.0804**	**123.0798**	-5.2
C8 H9 O $^+$		**121.0648**	**121.0647**	-0.8

Table S3. Ion species corresponding to the accurate monoisotopic *m/z* of dihydro-sulfo- gambierone with mass differences (Δppm) between theoretical monoisotopic mass and measured *m/z*. The shared ions with a delta of 2 Da between sulfo-gambierone and dihydro-sulfo-gambierone are shown in blue and ions specific to the new dihydro-sulfo-gambierone are shown in orange.

Ion formula	Ion	Theoritical monoisotopic mass	Measured *m/z*	Δ (ppm)
C51 H78 O22 S2 + NH4+	M+NH4+	**1124.4764**	**1124.4716**	-4.3
C51 H76 O22 S2 + H+	M+H+	**1107.4499**	**1107.4469**	-2.7
C51 H74 O21 S2 + H+	M-H2O+H+	**1089.4393**	**1089.4299**	-8.6
C51 H72 O20 S2 + H+	M-2H2O+H+	**1071.4288**	**1071.4169**	-11.1
C51 H76 O19 S + NH4+	M-SO3+NH4+	**1044.5196**	**1044.5176**	-1.9
C51 H76 O19 S + H+	M-SO3+H+	**1027.4931**	**1027.4888**	-4.2
C51 H74 O18 S + H+	M-SO3-H2O+H+	**1009.4825**	**1009.4773**	-5.2
C51 H72 O17 S + H+	M-SO3-2H2O+H+	**991.4719**	**991.4662**	-5.7
C51 H70 O16 S + H+	M-SO3-3H2O+H+	**973.4614**	**973.4540**	-7.6
C51 H76 O16 + NH4+	M-2SO3+NH4+	**964.5628**	**964.5605**	-2.4
C51 H76 O16 + H+	M-2SO3+H+	**947.5363**	**947.5330**	-3.5
C51 H75 O15+	M-2SO3-H2O+H+	**929.5257**	**929.5210**	-5.1
C51 H73 O14+	M-2SO3-2H2O+H+	**911.5151**	**911.5113**	-4.2
C51 H71 O13+	M-2SO3-3H2O+H+	**893.5046**	**893.5012**	-3.8
C51 H69 O12+	M-2SO3-4H2O+H+	**875.4940**	**875.4904**	-4.1
C51 H67 O11+	M-2SO3-5H2O+H+	**857.4834**	**857.4797**	-4.3
C45 H69 O14+		**833.4682**	**833.4647**	-4.2
C45 H67 O13+		**815.4576**	**815.4541**	-4.3
C45 H65 O12+		**797.4471**	**797.4438**	-7.4
C45 H63 O11+		**779.4365**	**779.4365**	-3.8
C43 H63 O11 +		**755.4365**	**755.4344**	-2.8
C43 H61 O10 +		**737.4259**	**737.4227**	-4.3
C43 H59 O9 +		**719.4154**	**719.4064**	-12.5
C38 H55 O8 +		**627.3891**	**627.3852**	-6.2
C38 H53 O7 +		**609.3786**	**609.3766**	-3.3
C38 H53 O6 +		**591.3680**	**591.3635**	-7.6
C18 H25 O3 +		**289.1798**	**289.1799**	0.3
C18 H23 O2+		**271.1693**	**271.1688**	-1.8
C15 H21 O2+		**233.1536**	**233.1537**	0.4
C10 H11 O2 +		**163.0754**	**163.0757**	1.8
C8 H11 O +		**123.0804**	**123.0800**	-3.2
C8 H9 O +		**121.0648**	**121.0649**	0.8
C7 H9 +		**93.0699**	**93.0696**	-3.2

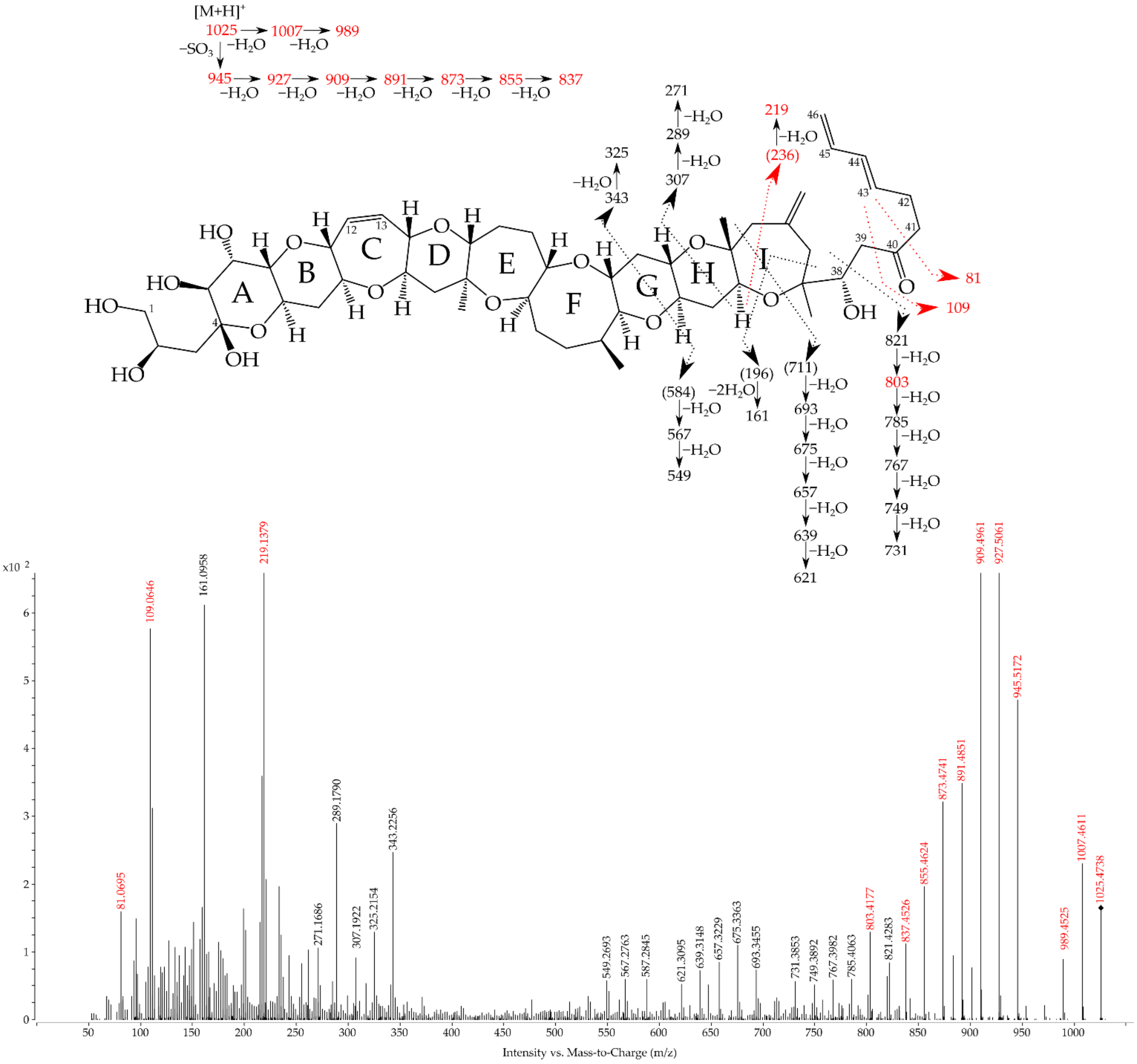

Figure S9. Targeted MS/MS fragmentation spectrum at a collision energy of 30 eV acquired in positive ESI mode on the precursor *m*/*z* 1025.4774 ($[M+H]^+$) with proposed fragmentation pathways. The *m*/*z* already reported in the literature for gambierone are shown in red [1,2], the proposal of new fragmentation pathways are shown in black.

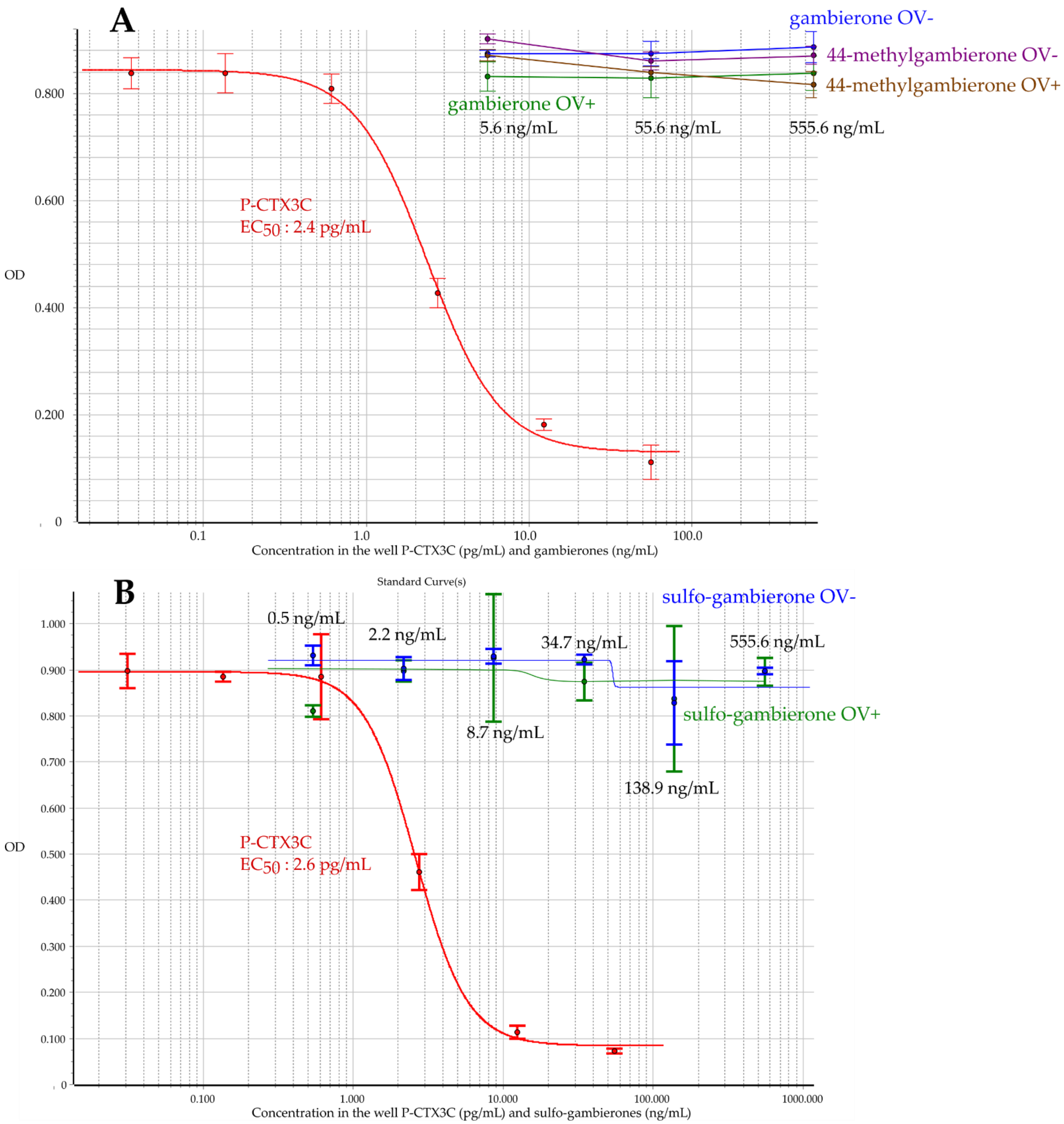

Figure S10. Neuro2a cell-based assay sigmoid dose-response curves (obtained with MARS 4.00R2 software) of P-CTX3C with O/V treatment (in red) from 0.031 to 55.56 pg mL^{-1} in the well with corresponding EC50 and **(A)** responses at three concentrations of gambierone with O/V treatment (in green) or without O/V treatment (in blue) and response of 44-methylgambierone with O/V treatment (in brown) or without O/V treatment (in purple) and **(B)** responses of the serial dilution of sulfo-gambierone (from 0.5 to 555.6 ng/mL) with O/V treatment (in green) or without O/V treatment (in blue).

Culture of Gambierdiscus excentricus *at Ifremer, Phycotoxin laboratory (Nantes, France)*

The two *Gambierdiscus excentricus* strains (Bahamas Gam 5 and Pulley-ridge Gam 2 [3]) were cultivated either in 1 L Fernbach or 225 cm^2 plastic flasks (Corning, CellBIND, Grosseron SAS, Coueron, France) filled with 300 mL of L1-Si medium (i.e. natural seawater from the English Channel, salinity 34.5, sterilized and then enriched with L1 nutrients except silica [4]). Cultures were maintained at 25 °C with a light intensity between 70 and 90 μmol m^{-2} s^{-1} provided by fluorescent tubes (cool-white and pink, Osram, Munich Germany) under a 12:12 h light:dark cycle. Cells were inoculated at a concentration of 500 cells mL^{-1} and harvested after three weeks at ca. 1800-2300 cells mL^{-1}. Harvesting was carried out using a 20 μm sieve to reduce the volume and cells were subsequently transferred into 50 mL Falcon tubes with sterile seawater. Cell pellets were obtained by centrifugation at 3500 *g* for 4 min and stored at -20 °C, after careful removal of supernatant and freeze dried to ensure a better stability.

Culture of Gambierdiscus excentricus *at NOAA Laboratory (Beaufort, NC, USA)*

Isolates were cultured, maintained, and collected as described in Litaker et al. (2017) [3]. Briefly, cells were cultured in a Percival Scientific incubator (Perry, IA, USA) maintained at 27 ˚C with a 12:12 h light:dark cycle. Photosynthetically active radiation (PAR) was maintained at 90–100 μmol photons m^{-2} s^{-1} (Full Spectrum Solutions, Jackson, MI, USA). Growth medium consisted of 0.2 μm filtered Gulf Stream seawater (salinity 33) in 250 mL tissue culture flasks with vented caps (BD Biosciences, Bedford, MA, USA). Vitamins and nutrients were added according to a modified K-medium protocol [5,6]. Cells were counted every three to four days using a Beckman Coulter Multisizer™ 3 particle counter (Beckman Coulter Inc., Brea, CA) equipped with a 280 μm aperture. Samples were mixed thoroughly to ensure the cells were evenly distributed prior to counting [5]. Cell densities were maintained at relatively low levels (250 to 1000 cells mL^{-1}) to avoid nutrient or CO_2 limitation. Cultures were diluted with fresh medium as needed to maintain cells in continual log phase growth [5]. Cells were harvested on a 20 μm sieve and washing them with filtered seawater into a 50 mL centrifuge tube. The cells were pelleted using centrifugation at 3200 *g* for 10 min, the supernatant carefully decanted, and stored at –20 ˚C until shipment [3].

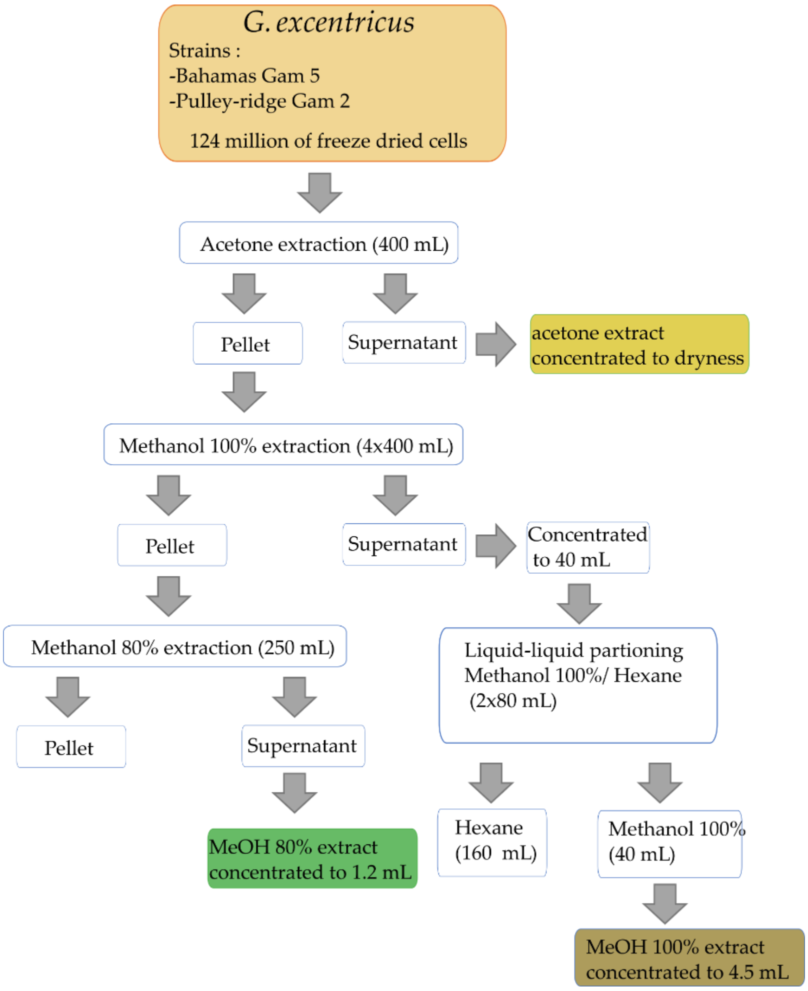

Figure S11. Cell extraction procedure used for biomass selective extraction

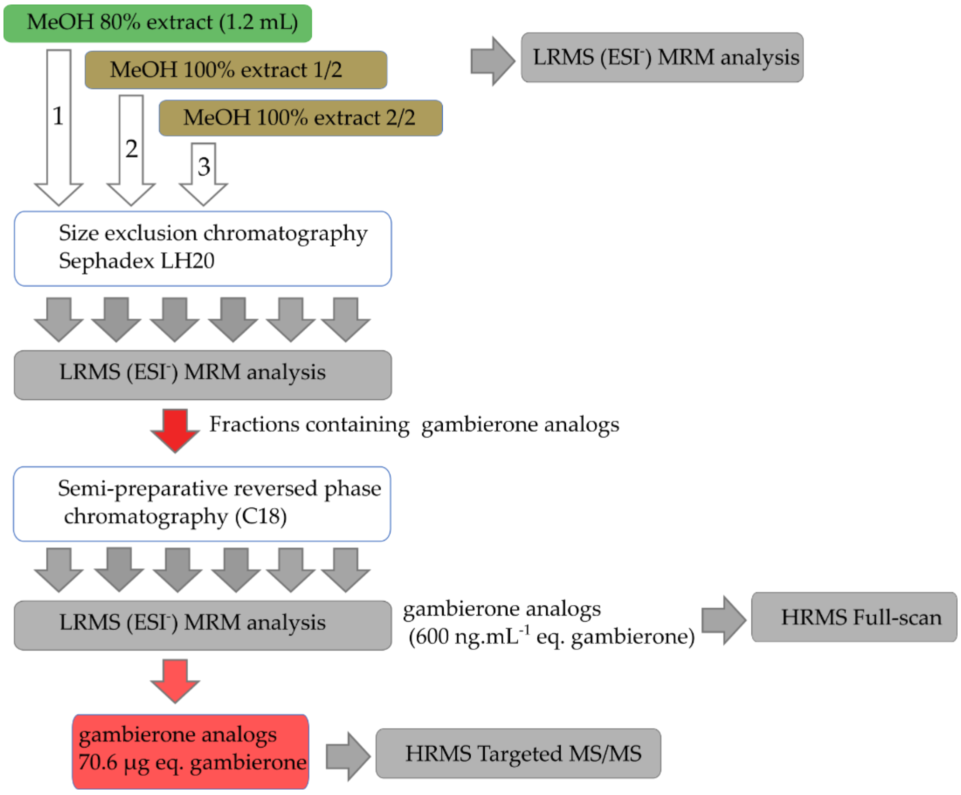

Figure S12. Fractioning and purification procedure for gambierone analogs isolation.

References

1. Estevez, P.; Sibat, M.; Leão-Martins, J.; Tudó, À.; Rambla-Alegre, M.; Aligizaki, K.; Diogène, J.; Gago-Martinez, A.; Hess, P. Use of Mass Spectrometry to Determine the Diversity of Toxins Produced by Gambierdiscus and Fukuyoa Species from Balearic Islands and Crete (Mediterranean Sea) and the Canary Islands (Northeast Atlantic). *Toxins* **2020**, *12*, 305, doi:https://doi.org/10.3390/toxins12050305.
2. Rodriguez, I.; Genta-Jouve, G.; Alfonso, C.; Calabro, K.; Alonso, E.; Sanchez, J.A.; Alfonso, A.; Thomas, O.P.; Botana, L.M. Gambierone, a Ladder-Shaped Polyether from the Dinoflagellate Gambierdiscus belizeanus. *Organic Letters* **2015**, *17*, 2392-2395, doi:https://doi.org/10.1021/acs.orglett.5b00902.
3. Litaker, R.W.; Holland, W.C.; Hardison, D.R.; Pisapia, F.; Hess, P.; Kibler, S.R.; Tester, P.A. Ciguatoxicity of Gambierdiscus and Fukuyoa species from the Caribbean and Gulf of Mexico. *PLoS One* **2017**, *12*, e0185776, doi:https://doi.org/10.1371/journal.pone.0185776.
4. Guillard, R.R.L.; Hargraves, P.E. Stichochrysis immobilis is a diatom, not a chrysophyte. *Phycologia* **1993**, *32*, 234-236, doi:https://doi.org/10.2216/i0031-8884-32-3-234.1.
5. Ransom Hardison, D.; Sunda, W.G.; Wayne Litaker, R.; Shea, D.; Tester, P.A. Nitrogen limitation increases brevetoxins in *karenia brevis* (*dinophyceae*): implications for bloom toxicity. *Journal of Phycology* **2012**, *48*, 844-858, doi:https://doi.org/10.1111/j.1529-8817.2012.01186.x.
6. Keller, M.D. Factors significant to marine dinoflagellate culture. *Toxic Dinoflagellates* **1985**, 113-116.
